# Supplementary material for: Towards Precision Sports Nutrition for Endurance Athletes: A Scoping Review of Application of Omics and Wearables Technologies
Source: Nutrients. 2024 Nov 19;16(22):3943. doi: 10.3390/nu16223943 (PMC11597302; doi:10.3390/nu16223943)
Supplement: Supplementary file 1 [file nutrients-16-03943-s001.zip › Supplementary File.pdf]

Table S1: Preferred Reporting Items for Systematic reviews and Meta-Analyses extension for Scoping Reviews (PRISMA-ScR) Checklist

SECTION	ITEM	PRISMA-ScR CHECKLIST ITEM	REPORTED ON PAGE #
**TITLE**			
Title	1	Identify the report as a scoping review.	**1**
**ABSTRACT**			
Structured summary	2	Provide a structured summary that includes (as applicable): background, objectives, eligibility criteria, sources of evidence, charting methods, results, and conclusions that relate to the review questions and objectives.	**1**
**INTRODUCTION**			
Rationale	3	Describe the rationale for the review in the context of what is already known. Explain why the review questions/objectives lend themselves to a scoping review approach.	**2-3**
Objectives	4	Provide an explicit statement of the questions and objectives being addressed with reference to their key elements (e.g., population or participants, concepts, and context) or other relevant key elements used to conceptualize the review questions and/or objectives.	**3**
**METHODS**			
Protocol and registration	5	Indicate whether a review protocol exists; state if and where it can be accessed (e.g., a Web address); and if available, provide registration information, including the registration number.	**/**
Eligibility criteria	6	Specify characteristics of the sources of evidence used as eligibility criteria (e.g., years considered, language, and publication status), and provide a rationale.	**3-4**
Information sources*	7	Describe all information sources in the search (e.g., databases with dates of coverage and contact with authors to identify additional sources), as well as the date the most recent search was executed.	**3**
Search	8	Present the full electronic search strategy for at least 1 database, including any limits used, such that it could be repeated.	**3 and Supplementary File, Table S2**

SECTION	ITEM	PRISMA-ScR CHECKLIST ITEM	REPORTED ON PAGE #
Selection of sources of evidence†	9	State the process for selecting sources of evidence (i.e., screening and eligibility) included in the scoping review.	**3**
Data charting process‡	10	Describe the methods of charting data from the included sources of evidence (e.g., calibrated forms or forms that have been tested by the team before their use, and whether data charting was done independently or in duplicate) and any processes for obtaining and confirming data from investigators.	**4**
Data items	11	List and define all variables for which data were sought and any assumptions and simplifications made.	**4**
Critical appraisal of individual sources of evidence	12	If done, provide a rationale for conducting a critical appraisal of included sources of evidence; describe the methods used and how this information was used in any data synthesis (if appropriate).	**4 and Supplementary File, Table S3**
Synthesis of results	13	Describe the methods of handling and summarizing the data that were charted.	**5**
**RESULTS**			
Selection of sources of evidence	14	Give numbers of sources of evidence screened, assessed for eligibility, and included in the review, with reasons for exclusions at each stage, ideally using a flow diagram.	**4-5**
Characteristics of sources of evidence	15	For each source of evidence, present characteristics for which data were charted and provide the citations.	**5-6**
Critical appraisal within sources of evidence	16	If done, present data on critical appraisal of included sources of evidence (see item 12).	**32 and Supplementary File, Table S3**
Results of individual sources of evidence	17	For each included source of evidence, present the relevant data that were charted that relate to the review questions and objectives.	**7- 10, 12-20, 22-26, 28-31**
Synthesis of results	18	Summarize and/or present the charting results as they relate to the review questions and objectives.	**6, 11, 21, 27**
**DISCUSSION**			
Summary of evidence	19	Summarize the main results (including an overview of concepts, themes, and types of evidence available),	**30-33**

SECTION	ITEM	PRISMA-ScR CHECKLIST ITEM	REPORTED ON PAGE #
		link to the review questions and objectives, and consider the relevance to key groups.	
Limitations	20	Discuss the limitations of the scoping review process.	**35**
Conclusions	21	Provide a general interpretation of the results with respect to the review questions and objectives, as well as potential implications and/or next steps.	**35-36**
**FUNDING**			
Funding	22	Describe sources of funding for the included sources of evidence, as well as sources of funding for the scoping review. Describe the role of the funders of the scoping review.	**36**

JBI = Joanna Briggs Institute; PRISMA-ScR = Preferred Reporting Items for Systematic reviews and Meta-Analyses extension for Scoping Reviews.
* Where *sources of evidence* (see second footnote) are compiled from, such as bibliographic databases, social media platforms, and Web sites.
† A more inclusive/heterogeneous term used to account for the different types of evidence or data sources (e.g., quantitative and/or qualitative research, expert opinion, and policy documents) that may be eligible in a scoping review as opposed to only studies. This is not to be confused with *information sources* (see first footnote).
‡ The frameworks by Arksey and O'Malley (6) and Levac and colleagues (7) and the JBI guidance (4, 5) refer to the process of data extraction in a scoping review as data charting.
§ The process of systematically examining research evidence to assess its validity, results, and relevance before using it to inform a decision. This term is used for items 12 and 19 instead of "risk of bias" (which is more applicable to systematic reviews of interventions) to include and acknowledge the various sources of evidence that may be used in a scoping review (e.g., quantitative and/or qualitative research, expert opinion, and policy document).

From: Tricco AC, Lillie E, Zarin W, O'Brien KK, Colquhoun H, Levac D, et al. PRISMA Extension for Scoping Reviews (PRISMAScR): Checklist and Explanation. Ann Intern Med. 2018;169:467–473. doi: 10.7326/M18-0850.

Table S2: Key search terms and search strategy

SEARCH TERMS	#
"personalised nutrition" OR "precision nutrition" OR "personalised sports nutrition" OR "precision sports nutrition" OR "sports nutrition" OR "nutrition" OR "digital twin	#1
"athlete" OR "endurance athlete*" OR "cyclist*" OR "runner*" OR "triathlete*" OR "ironman" OR "swimmer*" OR "rower*" OR "racewalker*" " OR "marathon" OR "long-distance" OR "cycling" OR "swimming" OR "cross-country" OR "running* OR "rowing"	#2
"systems biology" OR "omics*" OR "sportsomic*" OR "multiomic*" OR "nutrigenetic*" OR "nutrigenomic*" OR "metagenomic*" OR "microbiome" OR "proteomic*" OR "metabolomic*" OR "epigenetic*" OR "epigenomic*" OR "transcriptomic*" OR "continuous glucose monitor*" OR "wearable" OR "real-time sensor*" OR "smartwatch*"	#3
#1 AND #2 AND #3	#4
#1 AND #2 AND #3 AND (English[Filter]))	#5
**SEARCH DETAILS PUBMED**	**RESULTS**
(("personalised nutrition"[All Fields] OR "precision nutrition"[All Fields] OR (("person s"[All Fields] OR "personable"[All Fields] OR "personableness"[All Fields] OR "personal"[All Fields] OR "personalisation"[All Fields] OR "personalise"[All Fields] OR "personalised"[All Fields] OR "personalising"[All Fields] OR "personality"[MeSH Terms] OR "personality"[All Fields] OR "personalities"[All Fields] OR "personality s"[All Fields] OR "personalization"[All Fields] OR "personalize"[All Fields] OR "personalized"[All Fields] OR "personalizes"[All Fields] OR "personalizing"[All Fields] OR "personally"[All Fields] OR "personals"[All Fields] OR "persons"[MeSH Terms] OR "persons"[All Fields] OR "person"[All Fields]) AND ("sport s"[All Fields] OR "sports"[MeSH Terms] OR "sports"[All Fields] OR "sport"[All Fields] OR "sporting"[All Fields]) AND ("nutrition s"[All Fields] OR "nutritional status"[MeSH Terms] OR ("nutritional"[All Fields] AND "status"[All Fields]) OR "nutritional status"[All Fields] OR "nutrition"[All Fields] OR "nutritional sciences"[MeSH Terms] OR ("nutritional"[All Fields] AND "sciences"[All Fields]) OR "nutritional sciences"[All Fields] OR "nutritional"[All Fields] OR "nutritionals"[All Fields] OR "nutritions"[All Fields] OR "nutritive"[All Fields])) OR (("precise"[All Fields] OR "precised"[All Fields] OR "precisely"[All Fields] OR "preciseness"[All Fields] OR "precises"[All Fields] OR "precision"[All Fields] OR "precisions"[All Fields]) AND ("sport s"[All Fields] OR "sports"[MeSH Terms] OR "sports"[All Fields] OR "sport"[All Fields] OR "sporting"[All Fields]) AND ("nutrition s"[All Fields] OR "nutritional status"[MeSH Terms] OR ("nutritional"[All Fields] AND "status"[All Fields]) OR "nutritional status"[All Fields] OR "nutrition"[All Fields] OR "nutritional sciences"[MeSH Terms] OR ("nutritional"[All Fields] AND "sciences"[All Fields]) OR "nutritional sciences"[All Fields] OR "nutritional"[All Fields] OR "nutritionals"[All Fields] OR "nutritions"[All Fields] OR "nutritive"[All Fields])) OR "sports nutrition"[All Fields] OR "nutrition"[All Fields] OR "digital twin"[All Fields]) AND ("athlete"[All Fields] OR "endurance athlete*"[All Fields] OR "cyclist*"[All Fields] OR "runner*"[All Fields] OR "triathlete*"[All Fields] OR "ironman"[All Fields] OR "swimmer"[All Fields] OR "marathon"[All Fields] OR "long-distance"[All Fields] OR "cycling"[All Fields] OR "swimming"[All Fields] OR "cross-country"[All Fields] OR "running*"[All Fields]) AND ("systems biology"[All Fields] OR "omics*"[All Fields] OR "multiomic*"[All Fields] OR "nutrigenetic*"[All Fields] OR "nutrigenomic*"[All Fields] OR "metagenomic*"[All Fields] OR "microbiome"[All Fields] OR "proteomic*"[All Fields] OR "metabolomic*"[All Fields] OR "epigenetic*"[All Fields] OR "epigenomic*"[All Fields] OR "transcriptomic*"[All Fields] OR "continuous glucose monitor*"[All Fields] OR "wearable"[All Fields] OR "real time sensor*"[All Fields] OR "smartwatch*"[All Fields])) AND (english[Filter])	**435**
**SEARCH DETAILS WEB OF SCIENCE**	
((((((((((ALL=(athlete)) OR ALL=(endurance athlete)) OR ALL=(cyclist)) OR ALL=(runner)) OR ALL=(marathon runner)) OR ALL=(long-distance runner)) OR ALL=(triathlete)) OR ALL=(ironman triathlete)) OR ALL=(swimmer)) OR ALL=(ultra-marathon runner)) OR ALL=(trail runner)) OR ALL=(cross-country) AND (((((ALL=(personalised nutrition)) OR ALL=(precision nutrition)) OR ALL=(precision sports nutrition)) OR ALL=(personalised sports nutrition)) OR ALL=(sports nutrition)) OR ALL=(digital twin) AND ((((((((((((ALL=(wearable)) OR ALL=(real-time sensor)) OR ALL=(continuous glucose monitor)) OR ALL=(smartwatch)) OR ALL=(systems biology)) OR ALL=(omic)) OR ALL=(nutrigenetic)) OR ALL=(nutrigenomic)) OR ALL=(metagenomic)) OR ALL=(proteomic)) OR ALL=(transcriptomic)) OR ALL=(epigenomic)) OR ALL=(metabolomic)) OR ALL=(microbiome)) OR	**222**

ALL=(sportsomic) AND and Book Chapters or Retracted Publication or Meeting Abstract (Exclude – Document Types) and English (Languages)	
**SEARCH DETAILS SCOPUS**	
(athlete OR "endurance athlete" OR cyclist OR runner OR "marathon runner" OR "long-distance runner" OR triathlete OR "ironman triathlete" OR swimmer OR rower OR "ultra-marathon runner" OR "trail runner" OR "cross-country") AND ("personalised nutrition" OR "precision nutrition" OR "precision sports nutrition" OR "personalised sports nutrition" OR "sports nutrition" OR "digital twin") AND (wearable OR "real-time sensor" OR "continuous glucose monitor" OR smartwatch OR "systems biology" OR omic OR nutrigenetic OR nutrigenomic OR metagenomic OR proteomic OR transcriptomic OR epigenomic OR metabolomic OR microbiome OR sportsomic)	**354**

Table S3: Assessment of methodological quality of studies using PEDro scale

Study	Randomization	Allocation concealed	Baseline criteria comparable	Subjects blinded	Therapist blinded	Assessor blinded	Minimum of 85% at follow-up	Intention to treat analysis	Between-group statistics	Point and variance data	Total
Nelson et al., 2012 [37]	Y	N	Y	Y	Y	Y	Y	Y	Y	Y	9/10
Nieman et al., 2012 [39]	Y	Y	Y	N	N	N	Y	Y	Y	Y	7/10
Womack et al., 2012 [30]	Y	U	U	Y	Y	Y	Y	N	Y	Y	7/10
Nelson et al., 2013 [38]	Y	N	Y	Y	Y	Y	Y	Y	Y	Y	9/10
Nieman et al., 2013 [40]	Y	Y	Y	Y	Y	Y	Y	N	Y	Y	9/10
Nieman et al., 2014 [41]	Y	Y	Y	N	N	N	Y	N	Y	Y	6/10
Nieman et al., 2015 [42]	Y	Y	Y	N	N	N	Y	Y	Y	Y	7/10
Pataky et al., 2016 [32]	Y	U	U	Y	Y	U	Y	Y	Y	Y	7/10
Guest et al., 2018 [33]	Y	U	U	Y	Y	U	Y	N	Y	Y	6/10
Moreno-Pérez et al., 2018 [56]	Y	N	Y	Y	Y	N	N	N	Y	Y	6/10
Nieman et al., 2018a [49]	Y	Y	Y	N	N	N	Y	N	Y	Y	6/10
Nieman et al., 2018b [50]	Y	Y	Y	N	N	N	Y	N	Y	Y	6/10
Nieman et al., 2019 [48]	Y	Y	Y	Y	Y	Y	N	Y	Y	Y	9/10
Carswell et al., 2020 [34]	Y	U	Y	Y	Y	U	Y	Y	Y	Y	8/10
Huang et al., 2020 [59]	Y	N	Y	Y	N	N	Y	Y	Y	Y	7/10
Lin et al., 2020 [60]	Y	N	Y	Y	Y	N	Y	Y	Y	Y	8/10
Nieman et al., 2020 [51]	Y	Y	Y	N	N	N	Y	N	Y	Y	6/10
Olsen et al., 2020 [43]	Y	N	Y	N	N	N	Y	Y	Y	Y	6/10
Stander et al., 2021 [44]	Y	N	N	Y	N	N	Y	Y	Y	Y	6/10
Guest et al., 2022 [35]	Y	U	U	Y	Y	U	Y	N	Y	Y	6/10
Tabone et al., 2022 [62]	Y	Y	Y	N	N	Y	N	N	N	Y	5/10
Gaskell et al., 2023 [63]	Y	Y	Y	Y	Y	U	U	U	Y	Y	7/10
Gorski et al. 2023 [47]	Y	N	Y	N	N	N	Y	Y	Y	Y	6/10
Gross et al., 2023 [64]	Y	Y	N	Y	Y	N	Y	Y	Y	Y	8/10
Jin et al., 2023 [45]	Y	N	Y	Y	N	N	Y	U	Y	Y	6/10
Li et al., 2023a [65]	Y	Y	Y	Y	N	U	Y	N	Y	Y	7/10
Nieman et al., 2023 [52]	Y	Y	Y	Y	Y	Y	Y	N	Y	Y	9/10
Jeppesen et al., 2024 [46]	Y	N	Y	Y	N	N	Y	N	Y	Y	6/10
Nieman et al., 2024a [53]	Y	Y	Y	Y	Y	Y	Y	N	Y	Y	9/10
Nieman et al., 2024b [55]	Y	N	Y	Y	Y	Y	Y	Y	Y	Y	9/10
Sakaguchi et al., 2024 [54]	Y	N	Y	Y	Y	Y	Y	Y	Y	Y	9/10

Abbreviations: Y, yes; N, no; U, unclear.
